# Facile Fabrication of Double-Layered Electrodes for a Self-Powered Energy Conversion and Storage System

**DOI:** 10.3390/nano10122380

**Published:** 2020-11-29

**Authors:** Seungju Jo, Nagabandi Jayababu, Daewon Kim

**Affiliations:** Department of Electronic Engineering, Institute for Wearable Convergence Electronics, Kyung Hee University, 1732 Deogyeong-daero, Giheung-gu, Yongin 17104, Korea; joseungju@khu.ac.kr (S.J.); nagabandi.jay@khu.ac.kr (N.J.)

**Keywords:** energy storage, energy conversion, supercapacitors, triboelectric nanogenerators, water-assisted oxidation, double-layered structure

## Abstract

An aluminum double-layered electrode (DE-Al) was successfully employed as two electrodes in a symmetrical supercapacitor (double-layered electrode symmetric SC (DE-SC)) and as a positive layer of a triboelectric nanogenerator (DE-TENG) with the aim of energy conversion and storage using a selfsame structured, self-powered flexible device. A facile water-assisted oxidation (WAO) process and metal sputtering after the WAO process can allow the electrodes to greatly improve the active surface area and the conductivity, leading to the enhancement of the electrochemical performances of a supercapacitor (SC). Particularly, this double-layered structure fabrication process is extremely less time-consuming and cost-effective. The electrochemical test of the proposed DE-Al was systematically conducted by cyclic voltammetry (CV), galvanostatic charge-discharge (GCD) and electrochemical impedance spectroscopy (EIS), along with the in-depth characterizations of the surface. From these studies, the DE-Al offers exceptional electrochemical properties compared with other structures, which were utilized as the electrodes in the polyvinyl alcohol/phosphoric acid (PVA/H_3_PO_4_) gel electrolyte. The improved performance apparently evidenced from the electrochemical tests of fabricated SC resulted from the enhanced electrical conductivity and large active surface area. The specific capacitance and cycle-life stability of the DE-SC were investigated by using a GCD analysis. Additionally, the EIS curves before and after stability test (for 3500 cycles) were obtained to prove the long-term endurance of DE-SC. A vertical contact and the separation mode of the TENG were also fabricated by using the same DE-Al as a positive layer and polydimethylsiloxane (PDMS) as a negative layer. Finally, the fabricated SC and TENG were successfully combined using a bridge rectifier to convert and store the mechanical energy as electrical energy. This simple design and facile fabrication of a double-layered-electrode-based structure is promising for the development of an energy conversion and storage device.

## 1. Introduction

Increasing energy consumption and the depletion of conventional energy resources are urging new and renewable energy alternatives. Unfortunately, most of the renewable resources cannot generate energy continuously; thus, energy storage devices are required for long-term use. Lithium-ion batteries (LIBs) and supercapacitors (SCs) have been widely used as energy storage devices. Particularly, SCs have gained appreciable attention as energy storage devices [1,2,3,4] owing to their inherent advantages, such as fast charging/discharging rates, longer life span and high power density [5,6,7]. Moreover, the SCs with an extremely low internal resistance can produce a lot of power in a short period of time. Above all, the SCs have many benefits over the batteries and fuel cells that have suffered from poor energy density problems. This ultra-high capacity of SCs is suitable for electronic devices and energy storage applications that require rapid charging. Consequently, great attempts have been made at achieving the high energy density in SCs that is very crucial for energy storage systems [8,9].

Supercapacitors store electrical energy in the form of the electrostatic charge accumulated at the interfaces of electrode/electrolyte [10,11,12,13]. Accordingly, the enlarged surface area of the electrodes and high electrical conductivity have been considered to be the key factors to improving the electrochemical performances of supercapacitors [14,15,16,17]. Therefore, the research on enhancing the surface conductivity of the electrodes along with increasing active surface area has been widely conducted to improve the electrochemical performance of SCs. As for the consideration of these issues, choosing the best performing materials [18,19,20] and increasing their effective contact areas [21,22,23,24,25] are very important for the enhancement of the performances of SCs. Above all, surface modifications with nanostructured morphology can improve capacity effectively. There are various types of processes adopted for the modification of the electrode surface [26,27]. However, most of them are difficult in nature and ineffective in terms of time and cost.

The combination of an energy storage device with a mechanical energy harvesting device can offer several advantages. On the one hand, it can effectively minimize the usage of an external power source to charge the energy storage device. On the other hand, when there is no power generated during the absence of the mechanical movement, the energy stored in the storage device can be utilized. As we know, various methods have been adapted to convert mechanical energy into electrical energy with the aid of diverse mechanisms. Piezoelectricity [28,29], triboelectricity and electromagnetics [30] are widely studied and utilized for the efficient conversion of mechanical energy into electrical energy. Particularly, the triboelectric nanogenerators (TENGs) have gained enormous attention owing to their advantages, including cost-effectiveness, light weight, ease of fabrication and high electrical outputs even at low force and low frequencies [31,32].

Generally, the TENGs generate electricity via the contact and separation of two materials with different electron affinities due to the triboelectrification and electrostatic induction. In brief, triboelectrification provides oppositely polarized charges on the surface of each material and electrostatic induction drives the transformation of mechanical energy into electrical energy [33,34,35]. Therefore, this technology has become promising in the area of mechanical energy harvesting.

In this work, we propose an energy conversion and storage system consisting of a double-layered electrode symmetric SC (DE-SC) as an energy storage device and a double-layered electrode triboelectric nanogenerator (DE-TENG) as an energy harvesting device. The water-assisted oxidation (WAO) process was used, which has an inherently nanostructured surface [36,37]. The additional metal deposition onto the oxidation layer was conducted to improve surface conductivity. This simplified structure with the surface modification of a double-layered electrode has numerous advantages. Aluminum (Al) is cost-effective material, and the WAO process is less time-consuming and more simple (no need for any sophisticated equipment). Accordingly, the nanograss-structured electrodes resulting from the WAO process can effectively enhance the surface area. Further, the deposited Cu layer after the WAO process can maintain porous surface morphology and improve surface conductivity.

An electrochemical test on a three-electrode system [38,39] was conducted to prove the performances of DEs. The electrical results of each electrode (Bare-Al, WAO-Al, DE-Al) were compared elaborately. The higher specific capacitance of DE was observed from galvanostatic charge-discharge (GCD) plots. Above all, the applicability of the best performing material DE-Al, for practical applications [40,41], was tested by fabricating a supercapacitor device using two films of the DE-Al electrode, PVA/H3PO4, as a gel electrolyte, with cellulose membranes as separators. With excellent capacity and stable conductivity, the proposed DE-Al-based supercapacitor exhibits potential applications in flexible, simple and energy storage devices. Finally, the symmetric supercapacitor was successfully combined with a TENG to store the electrical energy. The DE-TENG can act as a power source for charging the DE-SC. Accordingly, our study affords new opportunities for fabricating self-powered flexible devices with energy harvesting-storage systems in future.

## 2. Experimental Section

### 2.1. Synthesis of Double-Layered Electrodes (DEs)

The synthesis of double-layered electrodes (DEs) was based on water-assisted oxidation (WAO). Firstly, an Al foil attached to a Polyimide (PI) substrate was oxidized at 80 °C in deionized (DI) water for 60 min. Following that process, an oxidized Al_2_O_3_ layer can be obtained from Al. After that, the additional metal layer of Cu was deposited on to this oxidized Al layer by means of sputtering. The power level of sputtering was fixed at 100 W, and the working time was 45 min. The mass of the active material coated on the substrate was 1.0 mg.

### 2.2. Preparation of PVA/H3PO4 Electrolyte

The gel electrolyte was prepared by mixing 10 wt% polyvinyl alcohol (PVA) powder with de-ionized water. Then, phosphoric acid (H3PO4) of 1.2 g was added to the gel electrolyte and stirred for 2 h. Upon evaporation of excess water, the electrolyte solidified.

### 2.3. Preparation of Pouch-Type DE-SC Device

The positive/negative electrodes and separator were first soaked in electrolyte and then sealed with aluminum laminated film to fabricate a pouch-type SC. A hot sealer (MSK-250, MTI, Seoul, Korea) was utilized to seal the pouch-type SC device. The footprint area of the final device was 1 cm^2^.

### 2.4. Fabrication of DE-TENG

The DE-TENG was fabricated with the combination of an Al layer (WAO process) and a Cu layer (sputtered), along with a PDMS and bare Al. The typical synthesis process was as follows: firstly, commercially available Al tape was oxidized at 80 °C in deionized (DI) water for 60 min to obtain the oxidized Al layers. Subsequently, the Cu layer was coated on to this oxidized Al layer by means of sputtering. The power level of the sputtering and the working time were fixed at 100 W and 45 min, respectively. Finally, the device was constructed by means of the systematic assembly of an oxidized Al layer, a Cu layer, PDMS and bare Al with polyimide (PI) as a substrate.

### 2.5. Characterization of Materials

A field emission scanning electron microscope (HR FE-SEM, Carl Zeiss MERLIN, Oberkochen, Germany) equipped with an energy dispersive X-ray spectroscope (EDS) was employed to investigate the surface morphologies and elemental compositions of the synthesized materials.

### 2.6. Electrochemical Characterization

The electrochemical properties of all electrodes were investigated by using an IviumStat electrochemical instrument (HS Technologies, Gyeonggi, Korea) with cyclic voltammetry (CV), galvanostatic charge/discharge (GCD) and electrochemical impedance spectroscopy (EIS) in a three-electrode system. Bare-Al, WAO-Al and DE-Al acted as working electrodes; Pt wire was used as the counter electrode; and Ag/AgCl was taken as the reference electrode in PVA/H3PO4.

### 2.7. Coupling of the Energy Conversion and Storage Systems

A TENG, which can convert mechanical energy into electrical energy, was used in the device. Mechanical energy was created by the contact−separations generated from an electrodynamic shaker (Labworks Inc. LW139.138-40, California, U.S.). The unit device of SC was connected to the TENG, including a bridge rectifier with the aim of storing the energy. In brief, the output of the TENG was connected to the input terminals of bridge rectifier and output terminals of bridge rectifier were given to the SC device. With this, the harvested electrical energy was successfully stored in the SC.

### 2.8. Electrochemical Properties

The following equations were used to obtain specific/areal capacitance, energy density and power density.

(1) $$C_{p}=\frac{I\times \Delta t}{m\times \Delta V}$$

(2) $$C_{a}=\frac{I\times \Delta t}{a\times \Delta V}$$

(3) $$E=\frac{C\times \Delta V^{2}}{2}$$

(4) $$P=\frac{E}{\Delta t}$$

where, *Cp*, *Ca*, *E* and *P* are specific capacitance (F g^−1^), areal capacitance (mF cm^−2^), energy density (Wh kg^−1^ or Wh cm^−2^) and power density (W kg^−1^ or W cm^−2^), respectively. *I*, *a*, ∆*t* and ∆*V* are areal current density (mA cm^−2^), area (cm^2^), discharging time (s) and potential window (V), respectively.

## 3. Results and Discussion

**Figure 1:**
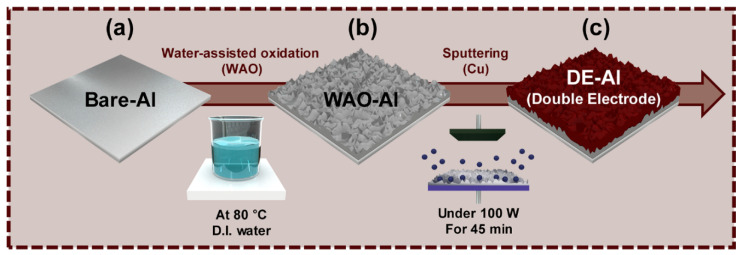
Schematic illustration and the fabrication procedure of each electrode: (**a**) Bare-Al; (**b**) water-assisted oxidation (WAO)-treated Al (WAO-Al); and (**c**) the double-layered electrode after Cu sputtering on the Al_2_O_3_ layer (DE-Al).

As shown in Figure 1, a double-layered electrode (DE) with aluminum (Al) as a current collector was facilely fabricated by a two-step method, which includes water assisted oxidation and metal sputtering. After preparing the bare Al substrate, the thin aluminum oxide layer shown in Figure 1a was formed by using the water-assisted oxidation (WAO) shown in Figure 1b; subsequently, the copper (Cu) layer was added, as shown in Figure 1c, with the aid of the RF sputtering. Accordingly, the fabricated device was finally constructed by means of the hierarchical assembly of Cu layer, a thin oxidized Al layer and a bare Al layer. Al, generally known as a cost-effective and good conductive material, has been gaining much attention from various fields. Additionally, the WAO process is a simple, less time-consuming and more cost-effective fabrication technique to create porous nanostructures on a metal’s surface than others. Furthermore, the deposition of an additional thin Cu layer on the thin Al_2_O_3_ layer arisen from the WAO process allows the substrate to drastically increase the conductivity of the surface, leading to maintaining the nano-porous structure on the surface. It is noteworthy that this nano-porous structure was fabricated with an elaborate hierarchical nanostructure without too much time or expensive being put into the fabrication process. This nano-porous structure can be utilized as an electrode of a supercapacitor, due to its high active area, which allows it to improve the electrochemical performance of the supercapacitor.

**Figure 2:**
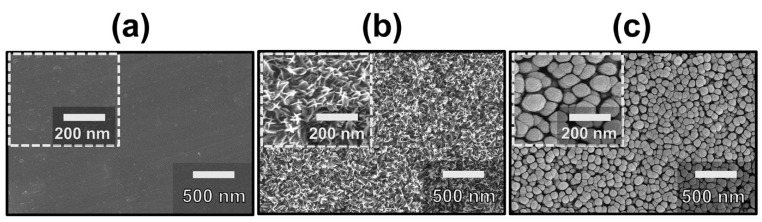
The FE-SEM images of surface morphologies: (**a**) Bare-Al; (**b**) WAO-Al; and (**c**) DE-Al.

To confirm the surface morphologies and elemental compositions of the surfaces of the fabricated electrodes, a scanning electron microscope (SEM) was utilized, as shown in Figure 2. Figure 2a shows the top-view SEM image of Bare-Al, confirming that the surface is flat without any porous-like morphology. Figure 2b exhibits the surface morphology of WAO-Al, which indicates the surface morphology was changed to a nanograss-structure. It is noted that the WAO process resulted in the formation of metal oxide nanostructures (Al_2_O_3_) on the metal surface (Al) with unique nanograss-like structures. The formation of Al_2_O_3_ on the Bare-Al can be proven from the EDS table, which shows two peaks corresponding to oxygen (O) and aluminum (Al). This type of nano-grass structure allows one to drastically increase its porosity, along with its highly active surface area, which plays a crucial role in the enhancement of the electrochemical performance. However, this WAO process comparatively decreased the conductivity of the surface arisen from the formation of the Al_2_O_3_ layer. Therefore, the conductivity of the surface should be increased without decreasing its high porosity to promote the electrochemical performance of the fabricated electrode. Another top-view SEM image shows the thin Cu layer was deposited on the WAO-Al by using RF. The metal sputtering by using Cu resulted in the formation of the final structure, i.e., a double-layered electrode. It is noteworthy that, by additional metal-deposition after the WAO process, the nano-grass structure shown in Figure 2b was transformed into a nano-pebble structure, as shown in Figure 2c.

Finally, the double-layered structure, which consists of the nano-pebble structure on the nano-grass structure, can be considered to effectively enhance the electrochemical performance due to its unique nanostructures. Below is the EDS table of the DE-Al, on which the three main elements correspond to Cu, Al and O—no impurities. It should be emphasized that this simple and easy fabrication process to obtain the DE-Al, thinly Cu coated WAO-Al, is able to improve the electrochemical performance by its unique structural properties with enhanced conductivity of the surface and the large active surface area of the fabricated electrode. Table 1 shows elemental composition of three samples (Bare-Al, WAO-Al, and DE-Al) from energy dispersive spectroscopy (EDS) analysis.

**Figure 3:**
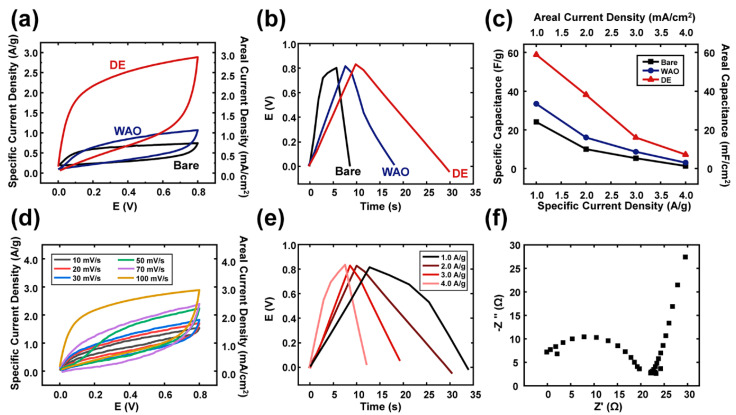
The electrochemical tests of each electrode via the three-electrode system. (**a**) The CV plots; (**b**) the galvanostatic charge-discharge (GCD) plots; and (**c**) the calculated specific/areal capacitance values. Additionally, the detailed electrochemical results for DE. (**d**) The CV plots under various scan rates; (**e**) the GCD curves with different applied current densities; and (**f**) the EIS curve of DE.

The electrochemical properties of Bare-Al, WAO-Al and DE-Al were evaluated with the aid of a three-electrode system. As shown in Figure 3a, the cyclic voltammetry (CV) plots of the fabricated electrodes within the potential window ranging from 0 to 0.8 V were collected at a scan rate of 100 mV/s. The quasi-rectangular shaped CV curves are demonstrated to compare the electrochemical properties for each electrode. It is evidenced from Figure 3a that the area enclosed by the CV curve of DE-Al is much higher than those of other two types of electrodes (Bare-Al and WAO-Al), indicating the superior performance of it. In addition, current responses were greater in the case of DE-Al compared to those of the other two-types of electrodes. It is considered that the increased active surface area and the increased conductivity are the main contributors for this enhanced performance. Its large active surface area improved the diffusion rate of electrolyte ions into the electrode, and its enhanced conductivity of the surface facilitated the rapid transportation of the electrons, which led to the improved overall electrochemical performance of the electrode. The quasi-rectangular shape of the CV curves indicates pseudocapacitive behavior, which originates from the Faradaic reactions (redox reactions) at the interface of the electrode and electrolyte.

Furthermore, all the GCD curves showed an approximatively triangular shape without an evident voltage fall from 0.8 V. Among the three types of electrodes, the DE-Al exhibited the longest discharging time, which was nearly 6 and 2.1 times longer than those of Bare-Al and WAO-Al, respectively (Figure 3b). It is noted that the synergistic affect arisen from the double-layered electrode which is the combination of Cu, Al_2_O_3_ and Al can hold more charge than other double-layered electrodes do. The enhanced conductivity and large porosity of the DE-Al-based electrode improve its reaction rate, which affects the electrochemical performance of the fabricated electrode.

Further analysis was conducted with the aid of discharging time of the prepared three types of electrodes. Figure 3c displays the calculated values of the specific/areal capacitances of three types of electrodes from the Equations (1) and (2), respectively. Under the conditions of various current densities ranging from 1.0 A/g to 4.0 A/g, the specific capacitance values of three electrodes were compared. It is notable that the DE-Al exhibited a larger value of specific capacitance than those of the other electrodes, which was ascribed to the enhanced active surface area and higher specific capacitance arisen from its double-layered structure of DE-Al. As well, it resulted in the aforementioned mechanism, in which the increased contact area promoted the diffusion of electrolyte ions and thereby improved the electrochemical performance.

As shown in Figure 3d, the CV curves were obtained from the DE-Al electrode at different scan rates of 10, 20, 30, 50, 70 and 100 mVs^−1^ in the steady voltage window between 0 and 0.8 V. When the scan rate was increased from 10 to 100 mVs^−1^, the corresponding current enhanced, while the shapes of CV curves remained more or less unchanged. All the CV curves similarly showed quasi-rectangular shape, indicating perfect pseudocapacitive behavior with redox reactions.

Additionally, the GCD plots acquired from different current densities of 1.0, 2.0, 3.0 and 4.0 A/g are shown in Figure 3e. Consequently, all the GCD curves maintained triangular shape without distinct voltage drops, which implies the DE-Al shows great capacitive performance. The electrochemical impedance spectroscopy (EIS) curves were utilized for the analysis of equivalent series resistance (ESR) of the DE-Al electrode. From the EIS curves, the intercepts of the *x*-axis give ESR values, and the diameters of the semicircles reveal the charge-transfer resistance (RCT) values of the electrodes.

As shown in Figure 3f, the RCT and ESR of DE-Al corresponded to approximately 20 ohm and 0.1 ohm, respectively. The relatively low values of the RCT and the ESR implied good electrochemical conductivity of the DE-Al electrode and offer superior electrochemical performance.

**Figure 4:**
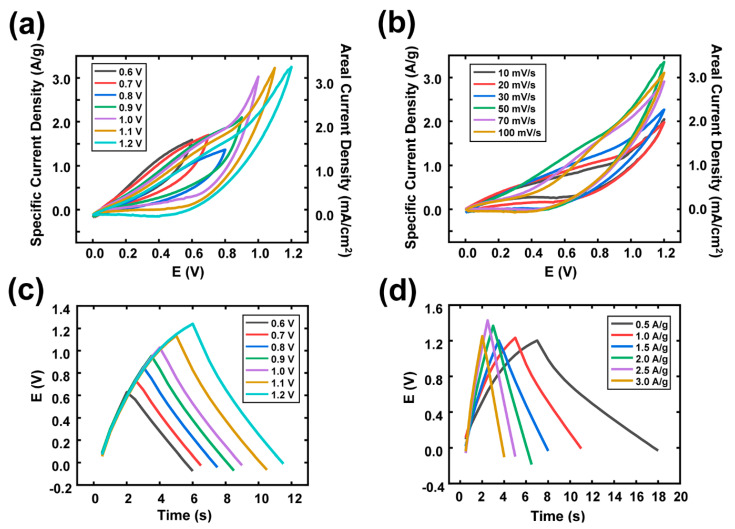
The electrochemical tests of the full device with the DE from the two-electrode system: The CV plots (**a**) under various potential windows and (**b**) under various scanning rates. The GCD curves plots (**c**) under various potential windows and (**d**) under different applied current densities.

To demonstrate the practical applicability of the DE-Al electrode, a flexible symmetrical supercapacitor device composed of two DE-Al electrodes and a H2SO4/PVA electrolyte was fabricated. The CV plots under various potential window and scanning rates are shown in Figure 4a,b.

The CV plots under various potential windows of 0.6, 0.7, 0.8, 0.9, 1.0, 1.1 and 1.2 V were obtained to optimize the potential window of the supercapacitor, as shown in Figure 4a. The distinct distortion of these curves did not exist, which confirmed great electrochemical performance of the fabricated supercapacitor using DE-Al electrodes. Until the potential window was increased from 0 to 1.2 V, the shape of the CV curves was not changed, indicating the voltage range from 0 to 1.2 V can be considered as the best suitable potential window for this DE-Al-based supercapacitor. Further, the depth analysis using CV was conducted under various scan rates, with the highest potential window being 1.2 V; see the CV curves shown in Figure 4a. The CV curves of the DE device showed its quasi-rectangular shape at the scan rates ranging from 10 to 100 mV s^−1^ as shown in Figure 4b.

As shown in Figure 4c, the GCD curves display nearly triangular shapes, indicating an ideal charging/discharging characteristic of the DE-Al supercapacitor. Even at the highest potential range of 1.2 V, the values of GCD curves did not show the saturation of voltage, or peculiar behavior. Subsequently, the GCD analyses under different current densities ranging from 0.5 to 3.0 A/g were conducted, as shown in Figure 4d. The triangle-shaped GCD curves were observed even at relatively high current densities without any distinct voltage drop, which implies that the DE-Al-based supercapacitor exhibited superior charging/discharging performance.

**Figure 5:**
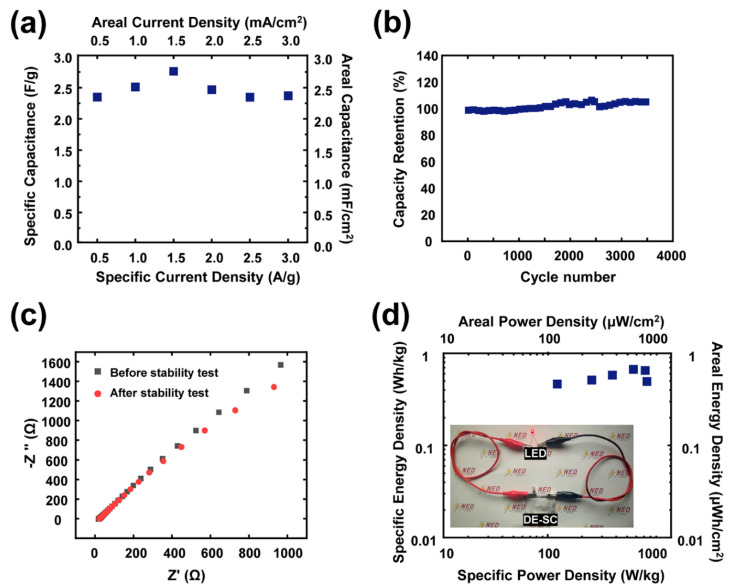
The electrochemical properties of the symmetric supercapacitor with DE. (**a**) The calculated specific capacitance and areal capacitance values. (**b**) The stability test for 3500 cycles from GCD curves and (**c**) the EIS curves before and after stability tests. (**d**) The energy and power densities of the symmetric DE-SC (inset image: showing the illumination of a commercial LED with the energy stored in DE-SC).

To further verify the charging/discharging performance of the DE-Al-based supercapacitor, the specific and areal capacitance values of full device were systematically calculated and graphically prepared, as shown in Figure 5a. In the two-electrode system, the highest specific capacitance of 2.8 F/g and areal capacitance of 2.8 mF/cm^2^ were observed at a current density of 1.5 A/g.

To confirm the long-term durability for practical applications, the cycling durability of the DE-Al-based supercapacitor was further investigated by using the GCD test, which revealed the capacitance retention of approximately 104.5% even after the charging/discharging operation of 3500 cycles, as shown in Figure 5b. Moreover, the values of EIS were nearly maintained at the initial value, i.e., the value before the charging/discharging operation without degradation of the electrochemical characteristic. Moreover, the EIS spectrum of the DE-Al-based supercapacitor indicated the small equivalent series resistance of 1.2 Ω, as shown in Figure 5c.

From the electrochemical analysis of DE-SC, the specific capacitance values are 2.29, 2.47, 2.8, 2.42, 2.28 and 2.3 F/g, corresponding to the various specific current densities of 0.5, 1.0, 1.5, 2.0, 2.5 and 3.0 A/g, respectively. Moreover, the specific energy density (*E*) and power density (*P*) are generally known as major criteria for evaluation of the supercapacitors. Energy density and power density are calculated from the Equations (3) and (4), respectively. The highest specific energy density of the device was 0.66 Wh/kg at a specific power density of 700 W/kg. The capability of the DE-SC device to illuminate a commercial LED was tested, as shown in the inset of Figure 5d. The energy stored in DE-SC can successfully light up the LED.

**Figure 6:**
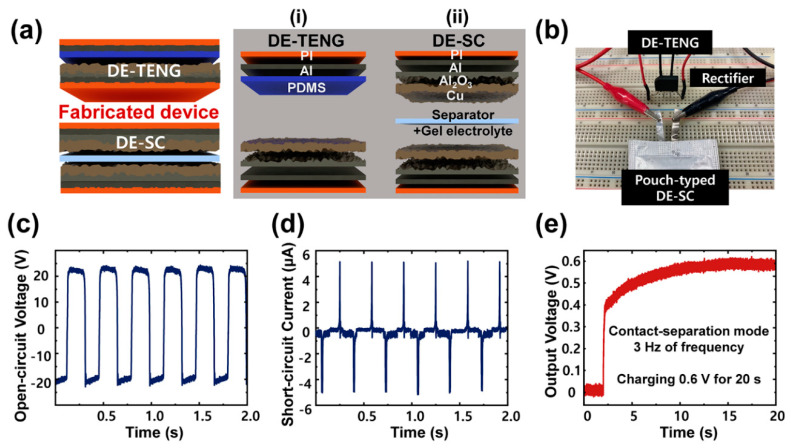
The device of self-powered energy conversion and storage we fabricated. (**a**) Schematic illustration of the device combining (i) the DE-TENG and (ii) the DE-SC. (**b**) Photograph of the circuit diagram with a rectifier, the DE-TENG and the DE-SC. (**c**) Open-circuit voltage and (**d**) short-circuit current of the DE-TENG. (**e**) Charging the DE-SC with the DE-TENG.

On the basis of the energy storage properties, the as-fabricated DE-SC was combined with the DE-TENG to manufacture a self-powered energy conversion and storage device. As shown in Figure 6a, the device is composed of an energy harvesting device (TENG part) and an energy storage device (SC part). The detailed structure of each part is demonstrated in Figure 6.

The bridge rectifier was connected to convert alternating current to direct current by transferring the energy provided by DE-TENG into electricity and storing it in the DE-SC (Figure 6b). With the friction of DE-TENG, the TENG converted the mechanical energy into electric energy and acted as an energy source for the SC. The electrical outputs, open-circuit voltage (*V*_oc_) and short-circuit current (*I*_sc_) of the DE-TENG were measured, as shown in Figure 6c,d.

During the periodical contact and separation of DE-TENG, an open-circuit voltage of 43 V and a short-circuit current of 5.1 μA were obtained from the 3 Hz of vibration frequency. For the endurance test, the value of the *V*_oc_ of the DE-TENG was highly stable at 32 V, even after 1 h (Supporting Information (SI) Figure S1). The electrical output generated by the TENG has to be stored to utilize it when the mechanical movement is absent. Therefore, the charging capability of the DE-TENG to charge the DE-SC was investigated by connecting the rectified output of the DE-TENG to a SC. As can be observed from Figure 6e, the generated electrical energy can be successfully stored in a SC through the rectifier. The voltage across a DE-SC was monitored in the charging process, and finally it could be charged to 0.6 V in about 20 s.

## 4. Conclusions

In summary, a double-layered electrode (DE) structure was successfully developed to enhance the electrochemical performances of a supercapacitor. The same DE electrode has been successfully employed as a triboelectric layer in a TENG. The unique surface modification by depositing a thin Cu layer on the Al_2_O_3_ layer arisen from the WAO process improved the conductivity and active surface area. The as-prepared DE-Al electrode exhibited superior performance—twice higher (calculated) specific capacitance than those of Bare-Al and WAO-Al. Further improvement in the capacitive performance can be realized by the symmetric SC device consisting of two DE films and H_3_PO_4_/PVA electrolyte. High cycling stability with great capacitance retention for 3500 cycles showed its feasibility. Moreover, the fabricated DE based symmetric SC has been successfully combined with the same DE-based TENG to make it a self-powered energy conversion and storage system. The fabricated device is capable of generating electrical energy from the mechanical energy via the TENG and storing it in the SC. Accordingly, the suggested device which is composed of a TENG and a SC can simultaneously harvest and store the energy to serve its needs. The fabrication process is simple, less time consuming and very cost effective (no sophisticated processes), and the structure is highly flexible and portable, which is very useful in powering wearable electronics. Therefore, this study surely emphasizes the need to fabricate self-powered and flexible devices with double-layered electrodes which are made by simple fabrication methods.

## Figures and Tables

**Table 1:** Results of the energy-dispersive X-ray spectroscopy (EDS) analysis of three samples (Bare-Al, WAO-Al and DE-Al).

Sample	Element (wt%)			Atomic (%)		
	Al	O	Cu	Al	O	Cu
Bare-Al	98.8	1.2		98.0	2.0	
WAO-Al	80.6	19.4		71.1	28.9	
DE-Al	31.7	5.8	62.5	46.6	14.4	39.0

## Supplementary Materials

The following are available online at https://www.mdpi.com/2079-4991/10/12/2380/s1, Figure S1: The long-term endurance test of DE-TENG for 1 h.
