# Supplementary material for: Facile Fabrication of Double-Layered Electrodes for a Self-Powered Energy Conversion and Storage System
Source: Nanomaterials (Basel). 2020 Nov 29;10(12):2380. doi: 10.3390/nano10122380 (PMC7760779; doi:10.3390/nano10122380)
Supplement: Supplementary file 1 [file nanomaterials-10-02380-s001.pdf]

Supplementary Materials

Facile Fabrication of Double-Layered Electrodes for a Self-Powered Energy Conversion and Storage System

1. The long-term endurance test of DE-TENG

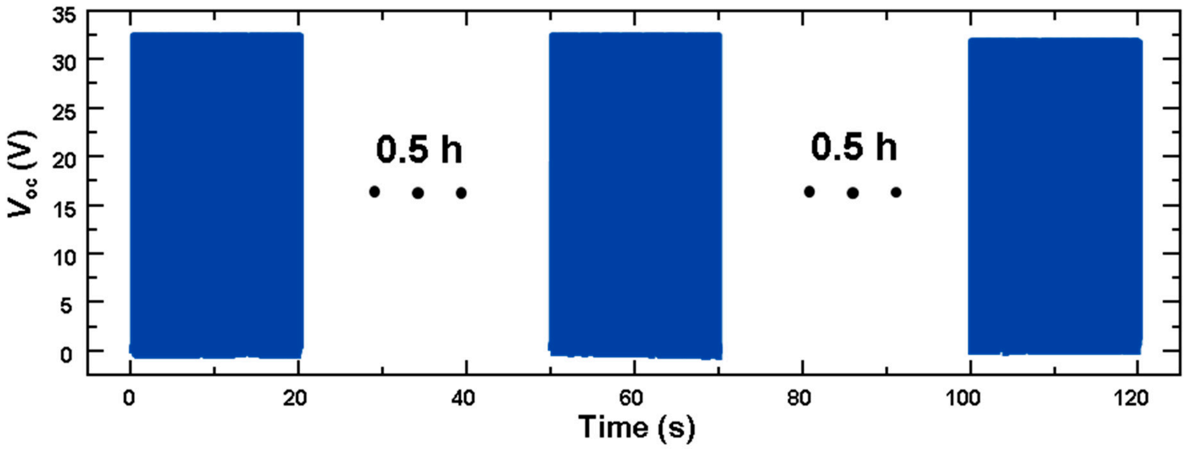

Figure S1. The long-term endurance test of DE-TENG for 1 h.
